# Use of Antimuscarinics in the Elderly

**DOI:** 10.1100/tsw.2009.55

**Published:** 2009-06-12

**Authors:** Bhavin Patel, Tamara Bavendam, Gopal Badlani

**Affiliations:** ^1^ Wake Forest University, Baptist Medical Center, Winston-Salem, NC, USA; ^2^ Pfizer Inc., New York, USA

**Keywords:** overactive bladder, elderly, anticholinergics

## Abstract

Overactive bladder (OAB) is a common, costly, and treatable condition in older persons. There is a wide array of available antimuscarinics for the treatment of these conditions; however, their side effect profile and the limited number of studies that evaluate their effect in the elderly curb their use. This review article focuses on OAB and its treatment, with special attention to the use of antimuscarinics in the elderly.

## EPIDEMIOLOGY AND BURDEN/IMPACT OF OVERACTIVE BLADDER

As the Baby Boomer population has aged, there has been a corresponding increase in the population that is elderly, those over 65. Unfortunately, with increasing age, the incidence of many ailments increases. This includes diseases of bladder storage and emptying. In essence, the bladder, sphincters, and urethra function together to allow for low-pressure filling and bladder storage, with complete emptying and continence. Pathology resulting in dysfunction of this normal storage and emptying is quite prevalent in the general population, with an increased prevalence in the elderly.

In 2006, the International Continence Society (ICS) defined overactive bladder (OAB) as “urgency, with or without urgency incontinence (UUI), usually with increased daytime frequency and nocturia” in the absence of other identifiable pathology[1]. The National Overactive Bladder Evaluation Program (NOBLE) investigated the prevalence of OAB in the U.S. population. Based on telephone interviews, they determined the overall prevalence of OAB to be 16.5%. The elderly, those over 65, were disproportionately affected, with a prevalence of OAB over 30%[2]. This is equivalent to approximately 10 million elderly individuals in the U.S.

The burden of OAB and UUI on the elderly can be devastating. Left untreated, the symptoms can lead to fear of wetting accidents[3], increased social isolation[3], depression[3], increased risk of falls and fractures[4], decline in health-related quality of life[4], and sleep disturbances with decreased daytime functioning[4]. The economic impact of OAB is also staggering. The estimated cost of OAB in 2003, including direct costs, nonreimbursable expenses, and indirect costs, was approximately $12 billion annually, a cost comparable to that associated with breast cancer[5].

Despite the significant impact of OAB on patients’ lives and the availability of treatment options, only a small percentage of elderly patients seek and receive treatment[6]. This is likely due to a number of factors, including under-reporting, polypharmacy, comorbidities, and difficulties in performing and interpreting urodynamic studies (UDS). In addition, the multifactorial nature of OAB in the elderly can make it seem as if intervention would not be successful even if recommended. In our experience, the elderly tend to be a population that under-reports their lower tract difficulties; this could be because they feel their problems may be a "normal" part of aging, are embarrassed by their symptoms, or do not know that noninvasive treatment options exist. Polypharmacy and comorbidities, such as decreased mobility, may make it more difficult to predict the effect and side effects of agents used in the medical treatment of bladder dysfunction, making some physicians hesitant to prescribe them. It is important for physicians to identify patients who are at risk for OAB and actively screen for OAB, particularly in the elderly. Early identification may help to identify specific causes and contributing factors that could make treatment more successful, and prevent worsening of OAB and its consequences.

## PATHOPHYSIOLOGY OF OAB

OAB symptoms can arise from one or more of the following pathologic mechanisms: patchy detrusor dennervation, smooth muscle hypertrophy, enhanced intracellular coupling, changes in C fiber density/responsiveness to stimuli, local change in muscarinic receptors, or improved sensory signaling (such as changes in suburothelial afferent response to bladder filling)[7,8]. The net effect of these changes is a trend toward inappropriate bladder contraction, leading to urgency, the hallmark of OAB, and increased micturition frequency. Decreased bladder sensation associated with nerve deterioration or medications may lead to incontinence associated with an involuntary detrusor contraction without the sensation of urgency or any sensation at all. Furthermore, these changes can be enhanced with increased urine production; thus, oral fluid–induced increased urine production and physiologic polyuria on recumbency, particularly in the elderly, can increase OAB symptomatology.

### Diagnosing OAB

A focused history and physical examination should be performed for all patients with symptoms of OAB. The history should focus on the frequency and timing of voiding; the presence of urgency, incontinence, dysuria, hematuria; any aggravating factors; fluid intake; comorbidity (including diabetes and constipation); and medication and supplement use. The physical exam should identify any neurologic or pelvic anomalies that may contribute to symptoms. A urinalysis should be done on all patients to assess for infection or hematuria. A postvoid residual is a simple and relatively low-cost procedure that should be done in those with OAB, as it may show surprising amounts of residual urine in a symptomless patient. It is important to avoid overinterpreting an isolated increased postvoid residual, as the patient may not be able to initiate a normal void in the office setting "on demand".

Reversible causes of urgency and incontinence should be sought and treated[9]. These include delirium, restricted mobility, urinary retention, lower urinary tract infection, diabetes, atrophic vaginitis, constipation and stool impaction, and polyuria due to excess fluid intake or volume overload. Additionally, a complete listing of prescription and nonprescription medications and supplements is crucial, as some of these agents, including narcotics, antidepressants, and diuretics, can have a profound impact on bladder sensation and voiding efficiency.

In evaluating symptoms of urgency and frequency in the elderly, one should keep in mind that there is a decrease in bladder contractility, sensation, and ability to postpone voiding with increasing age[10]. Additionally, there is an increase in postvoid volume, involuntary detrusor contraction, and sleep disorders with aging, all of which may worsen the symptoms of OAB[10,11]. Given these age-related changes, a UDS could be particularly helpful in determining the most appropriate treatment. However, physical and cognitive limitations can make UDS unfeasible in some elderly patients and difficult to interpret in others.

## MANAGEMENT OF OAB

The goal of OAB management is to restore socially acceptable urinary continence with minimal side effects. Many therapeutic options for the patient with OAB exist, including nonpharmacologic treatment, pharmacologic treatment, and surgical treatment. Nonpharmacologic treatment includes the use of behavioral interventions, such as bladder retraining, pelvic floor muscle exercises with or without biofeedback, dietary and fluid management, and medication management. These therapies alone or in combination with pharmacologic treatment have shown benefit in controlling symptoms associated with OAB[12,13].

Pharmacologic treatment includes antimuscarinics, membrane channel drugs, antidepressants, prostaglandin inhibitors, vasopressin analogs, direct muscle relaxants, and beta agonists[14]. Intravessical agents, such as Botulinum toxin, have also been efficacious in the treatment of OAB[14]. When other, less-invasive modalities have failed, surgical options, such as neural modulating devices, detrusor myectomy, and augmentation cystoplasty, may be considered[14]. From here in, the focus will be on the use of antimuscarinics.

## ANTIMUSCARINICS IN OAB

### Mechanism of Action

In OAB, the main target for pharmacologic therapy has been the muscarinic receptor. While nearly 80% of muscarinic receptors in the bladder are $M_2$, normal human detrusor contraction is mediated by the $M_3$ receptor. Additionally, the interaction between the urothelium-suburothelium, which in OAB excites afferents contributing to OAB symptoms, is mediated by acetylcholine and muscarinic receptors[15]. Antimuscarinics work to alleviate symptoms of OAB by blocking bladder muscarinic receptors, which increases the volume to the first involuntary contraction and total bladder capacity, and decreases the amplitude of the involuntary detrusor contraction[15]. Antimuscarinics available in the U.S. for the treatment of OAB include oxybutynin (Ditropan®), tolterodine (Detrol®), trospium (Sanctura®), darifenacin (Enablex®), solifenacin (Vesicare®), and fesoterodine (Toviaz™). All of these agents have a long-acting formulation and have demonstrated reduction in UUI and micturition episodes per 24 h.

### Side Effects

In addition to their location in the bladder, muscarinic receptors are found throughout the body serving varying functions. There are no antimuscarinic agents that are completely selective for the bladder. Nonbladder blockade of muscarinic receptors can have deleterious effects on these other organs were muscarinic receptors are present. Side effects include dry mouth, blurred vision, constipation, and gastroesophageal reflux.

Antimuscarinic side effects have the potential to occur in all patients, but may be more pronounced in the elderly. Dry mouth, a commonly reported side effect, may impair an elderly person's ability to speak and communicate, promote malnutrition, and increase oral fluid intake, which may worsen OAB symptoms. Blurred vision may lead to motor vehicle accidents or falls, which can lead to injury or serious fractures, particularly in those with reduced bone density. The effect of these agents on bowel motility in a population already predisposed to constipation may lead to fecal impaction and worsening OAB symptoms, including UUI.

The role of antimuscarinic medications on the central nervous system (CNS) is still up for debate. It is clear that muscarinic receptors are found in the brain ($M_1$ – $M_5$)[16]. Additionally, studies in mice show that these receptors play a pivotal role in learning, memory, control of movement, pain reception, and in regulation of the circadian cycle[17]. Functional studies in humans, after cholinergic blockade with scopolamine, have demonstrated decreased brain activity in areas associated with memory[18]. Additionally, there are measurable changes in EEG patterns with oxybutynin use[18]. Furthermore, studies show a detrimental effect of scopolamine on the memory, spatial recognition, and response speed that was more pronounced in an elderly cohort[18]. This enhanced effect in the elderly may be due to normal loss of CNS cholinergic receptors with age[18]. The potential block of these receptors can have deleterious effects. In the elderly, particularly those with mild cognitive impairment and medical comorbidity, these side effects can greatly affect a patient's independence and their compliance.

Only darifenacin has completed a prospective, randomized, placebo-controlled study in the elderly cohort[19]. Most other antimuscarinic studies have published post hoc analysis of the elderly cohort in some of their clinical trials.

## Specific Agents

- **Oxybutynin** is a nonselective, antimuscarinic, tertiary amine that is extensively metabolized in the liver by cytochrome p-450 (3A4). It is available in an immediate-release form, in an extended-release form, and as a patch or gel There are no specific studies that examine the use of this drug in an elderly cohort. The immediate-release form has been shown to be efficacious in reducing frequency, incontinence, and subjective symptoms of OAB[20]. However, side effects are prevalent with 73% of patients having dry mouth[21], and this along with constipation or blurred vision, led to a discontinuation of up to 25%[21]. The extended-release form is associated with 43% fewer side effects[22]. The transdermal form also has a lower incidence of side effects compared to the immediate-release form, but skin site reactions in up to 17% limit its use[23,24]. Oxybutynin has been shown to cause changes in EEG patterns, deficits in cognitive function tests, and even drowsiness, hallucinations, and psychosis[18]. The starting dose of the immediate-release form is 5 mg three times a day ($90/month), the extended-release form is 5 mg daily ($113/month), and the patch is dosed twice a week ($112/month).
- **Tolterodine** is a nonselective, tertiary amine that is metabolized in the liver by cytochrome p-450 (2D6) with an active metabolite 5-hydroxymethyl tolterodine. The immediate-release form is mostly excreted in the urine and has a half-life of 2–3 h. Tolterodine has low lipophilicity and is a large molecule, making it unlikely to cross an intact blood-brain barrier (BBB). While there are no specific studies that examine the use of this drug in the elderly cohort, there is published information on tolterodine and its use in the elderly. In a post hoc analysis of data for patients ≥65 years, Zinner and colleagues showed that tolterodine LA had a significant reduction of UUI compared to placebo[25]. In the post hoc analysis of the open-label IMPACT study, the elderly had statistically significant reduction in episodes of urgency, day- and night-time frequency, and UUI compared to baseline. In the Zinner analysis, the most common side effect was dry mouth, and it occurred in similar rates between the elderly and nonelderly (24%)[25]. The rate of constipation and CNS side effects of somnolence, headache, and dizziness were similar to placebo in the ≥65 and <65 years groups[25]. The incidence of cardiac events on tolterodine was similar to placebo[25]. While the recommended starting dose in all patients is 2 mg twice a day or 4 mg daily of the long-acting formulation, we typically start with the lower dose of 1 mg twice a day ($140/month) or 2 mg daily ($120/month), and increase the dose as needed.
- **Trospium** is a quaternary amine that has a 10% bioavailability, is excreted mostly in the feces, and has a half-life of 20 h. It has no muscarinic receptor subtype selectivity and is not metabolized by the cytochrome p-450 system. Trospium's low lipophilicity, large molecular weight, and polarity make it less likely to cross the BBB. There are no specific studies that examine the use of this drug in an elderly cohort. It has an efficacy similar to oxybutynin in a head-to-head comparison with bid dosing for both drugs, showing decreases in frequency of urination, frequency of incontinence, and urgency episodes. Trospium has a better tolerability than oxybutynin due to a decreased incidence of side effects[26]. The typical starting dose is 20 mg twice a day, which costs roughly $130/month.

- **Darifenacin** is a tertiary amine that is metabolized by the liver by cytochrome p-450 (2D6 and 3A), and excreted in the urine and feces. It has a half-life of 13–19 h. It is highly selective for the $M_3$ receptor. Darifenacin has been shown to be efficacious in the treatment of OAB in the elderly, with sustained improvement in symptoms at 2 years with good tolerability[27]. Furthermore, Chapple and colleagues showed that in a study of the elderly, there was an improvement in quality of life scores[19]. Additionally, studies have shown an increase in mean warning time with darifenacin[28]. Its molecular makeup makes it unlikely to cross the BBB (large molecule with positive polarity) and, thus, it is unlikely to have any effect on the CNS. In a recent study that examined its effects on cognition in a healthy elderly cohort, darifenacin did not result in significant differences in memory scanning, speed of choice recognition, and word recognition compared to placebo[29]. The typical starting dose is 7.5 mg of the extended-release form, costing approximately $121/month.
- **Solifenacin** is a tertiary amine that has a bioavailability of 90%, is metabolized by the liver by cytochrome p-450 (3A4), and is excreted in the urine. It has a half-life of 45–68 h. It has some selectivity for the $M_3$ receptor and is available in once-daily dosing. There are no specific studies that evaluate its use and side-effect profile in an elderly cohort, although pooled analysis of subjects >65 years in placebo-controlled trials have shown that solifenacin is efficacious for the treatment of OAB[30]. Its flexible dosing may make it more efficacious than tolterodine[19]; however, it has a higher rate of constipation than some of the other antimuscarinics, 9.1%[31]. The typical starting dose is 5 mg daily, with the possibility of increasing the dose to 20 mg, costing approximately $125/month.

## TREATING OAB IN THE ELDERLY

Combining the above information to make generalized recommendations on the use of antimuscarinic medications in the elderly is difficult, as each patient will present different challenges. Each patient has a different level of cognitive ability, different comorbidities and medications, as well as varying ability to tolerate medication side effects.

Our treatment protocol is started with nonpharmacologic therapy. This includes an initial focus on fluid management, searching for and treating reversible causes (including causative medications), and bladder training with biofeedback for pelvic floor muscle strengthening, as appropriate. If elderly patients continue to have persistent symptoms, we discuss the benefits and specific side effects of anticholinergic medications with them. They are then started on a selective anticholinergic with low potential to cross the BBB at low dose (such as tolterodine).

The patient is then re-evaluated in clinic in 1 week (once steady-state concentration is met). The patient or the caregiver is asked about improvement in OAB symptomatology and specific side effects are assessed. The focus is on dry mouth, constipation, and changes in mental status. If symptoms persist, but the medication is well tolerated, the dose is titrated to improve efficacy. If symptoms persist, but the medication is not well tolerated, the patient is given a washout period of 1 week and then started on an alternative oral anticholinergic, such as solifenacin or darifenacin. Specific side effects are proactively addressed. For example, if constipation is a problem, the patient can be placed on a stool softener or bowel management program, including the use of laxatives, as needed. Patients are advised to sip on water, suck on hard candy, or chew gum if they experience dry mouth. If this is still unsuccessful, they are evaluated for other therapy, including sacral modulation and Botulinum toxin.

## CONCLUSION

OAB is a common, costly, and treatable condition in older persons. While all currently available antimuscarinics for the treatment of OAB have included older persons in their clinical trials, few studies have been initiated specifically for the older cohort. Current data show that antimuscarinics can be efficacious in the elderly, with similar tolerability to younger persons. Concomitant medical conditions and multiple medications can make treatment more challenging. However, with appropriate setting of expectations and education regarding behavioral interventions, treatment can be very successful. In some patients, success may need to be defined as preventing worsening of the symptoms that could lead to nursing home placement.
